# Botulinum Toxin to Improve Results in Cleft Lip Repair: A Double-Blinded, Randomized, Vehicle-Controlled Clinical Trial

**DOI:** 10.1371/journal.pone.0115690

**Published:** 2014-12-26

**Authors:** Chun-Shin Chang, Christopher Glenn Wallace, Yen-Chang Hsiao, Chee-Jen Chang, Philip Kuo-Ting Chen

**Affiliations:** 1 Graduate Institute of Chemical and Materials Engineering, College of Engineering, Chang Gung University, Taoyuan, Taiwan; 2 Graduate Institute of Clinical Medical Sciences, School of Medicine, Chang Gung University, Taoyuan, Taiwan; 3 Craniofacial Research Center, Department of Medical Research, Department of Plastic Surgery, Chang Gung Memorial Hospital, Taoyuan, Taiwan; University of Toronto, Canada

## Abstract

**Background:**

Most patients with facial scarring would value even a slight improvement in scar quality. Botulinum toxin A is widely used to alleviate facial dynamic rhytides but is also believed to improve scar quality by reducing wound tension during healing. The main objective was to assess the effect of Botulinum toxin on scars resultant from standardized upper lip wounds.

**Methods:**

In this double-blinded, randomized, vehicle-controlled, prospective clinical trial, 60 consecutive consenting adults undergoing cleft lip scar revision (CLSR) surgery between July 2010 and March 2012 were randomized to receive botulinum toxin A (n = 30) or vehicle (normal saline; n = 30) injections into the subjacent orbicularis oris muscle immediately after wound closure. Scars were independently assessed at 6-months follow-up in blinded fashion using: Vancouver Scar Scale (VSS), Visual Analogue Scale (VAS) and photographic plus ultrasound measurements of scar widths.

**Results:**

58 patients completed the trial. All scar assessment modalities revealed statistically significantly better scars in the experimental than the vehicle-control group.

**Conclusion:**

Quality of surgical upper lip scars, which are oriented perpendicular to the direction of pull of the underlying orbicularis oris muscle, is significantly improved by its temporary paralysis during wound healing.

**Trial Registration:**

ClinicalTrials.gov NCT01429402

## Introduction

A cutaneous scar is the end result of a complex wound healing process that follows dermal injury [1], [2]. The characteristic shape, location and orientation of a cheiloplasty scar mark the patient for life as having been born with a cleft lip deformity. Such facial scars are conspicuous and difficult to conceal. Most patients with facial scarring harbor dissatisfaction and would value even a minor improvement [3].

The muscles of facial expression lie superficially, do not have bony attachments and create expressions by altering the tension of adjacent skin. However, skin tension that is perpendicular to an incision or laceration is known to increase the risk of unfavorable scarring as a result of the distracting forces exerted on the healing wound [4]. Therefore, reductions in facial muscular activity that result in decreased skin tension during healing may improve facial scarring. Gassner et al found that botulinum toxin injections tended to improve facial scars [5]. However, their study involved the use of different mechanisms of injury, suture materials and wound orientations, each of which is a factor known to affect scar quality independently [6]. Moreover, scar outcomes were only assessed subjectively.

**Figure 1:**
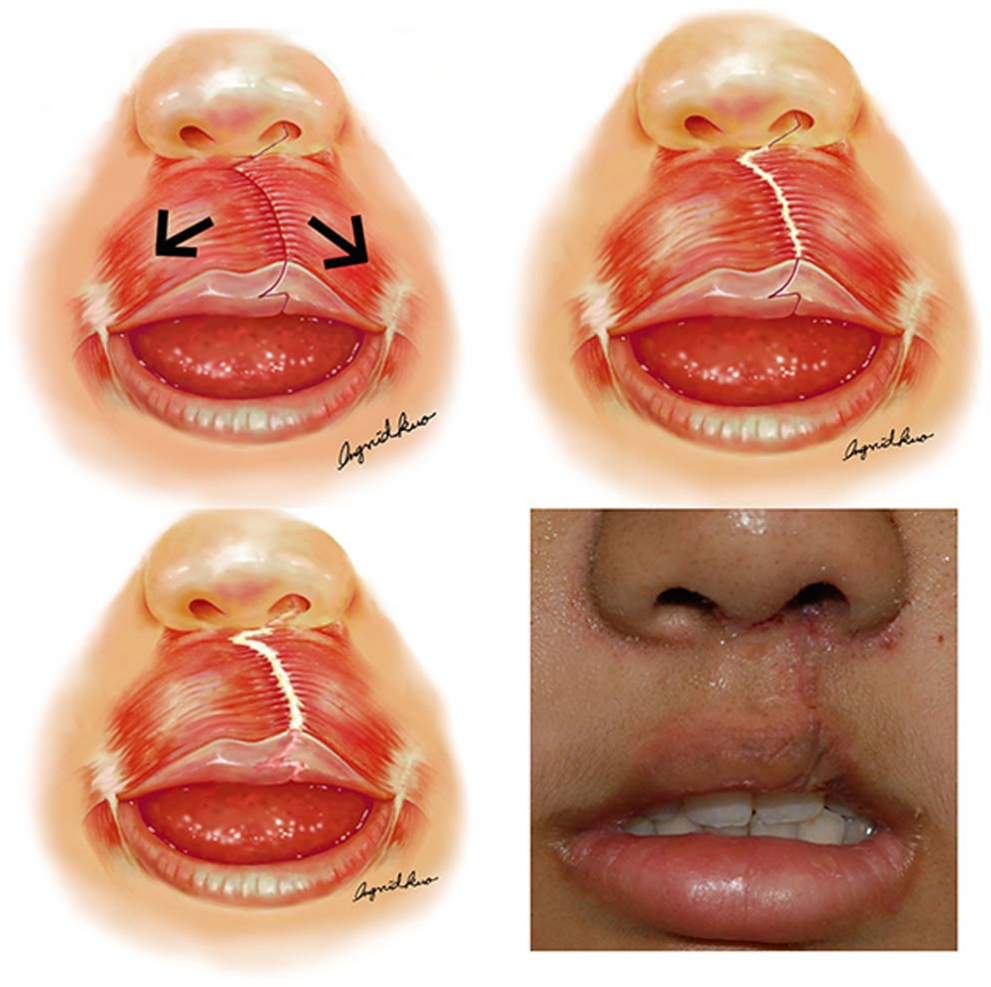
Contractions of the orbicularis oris muscle are believed to inflict repeating micro-trauma to the healing wound, causing an initially narrow scar to slowly widen. Lower right: A patient in the control group exhibiting an elevated scar after suture removal.

A standardized method of cleft lip scar revision (CLSR) has been established for more than a decade at our center. The design of incisions, sutures used and postoperative care are all protocolized. CLSR incisions are unavoidably perpendicular to the direction of pull of the underlying orbicularis oris muscle, which therefore exerts unfavorable forces across the sutured wound (Fig. 1). We routinely use taping to try to control against this pull, and routinely apply topical silicone sheeting, which is known to improve scarring [7]; however, despite these efforts, we still observe hypertrophic scarring following CLSR. There remains a need to improve scarring so that the lifelong stigma endured by patients of having obvious evidence of cleft lip repair can be reduced.

We hypothesized that temporary paralysis of the orbicularis oris muscle with botulinum toxin may improve the CLSR scar and tested this with a randomized, double-blinded, prospective, vehicle-controlled clinical trial. This is the first such trial that has evaluated the effects of botulinum toxin on scars from uniformly sustained, orientated, repaired and managed upper lip wounds in a single ethnic population.

## Methods

This was a randomized, double-blinded, prospective, vehicle-controlled, two-armed parallel clinical trial designed to investigate whether the injection of botulinum toxin into a perpendicularly oriented muscle (orbicularis oris) subjacent to a constant facial (CLSR) wound immediately after completion of surgery affected the quality of the resultant scar (S1 Protocol).

### Ethics

The trial was approved by the Institutional Review Board (IRB) of Chang Gung Memorial Hospital. After clear explanation of the study, an IRB approved written informed consent was provided by all participants. If patient was minor, the IRB approved written informed consent was provided by parents or guardians. The date at which the ethics committee approved the study was 16 October 2009, the date that patient recruitment started was 9 July 2010 and the date that follow-up completed for the final patient was 20 March 2012.

This study was registered at clinicaltrials.gov [ID NCT01429402]. Registration occurred after the trial began due to administrative difficulties with the registration process. The authors confirm that all ongoing and related trials for this drug are registered.

### Study Sample Size

The sample size was calculated based on the results of a pilot study. Ten consecutive outpatients underwent upper lip scar evaluation in March 2009 using the Vancouver Scar Scale (VSS) and presented a mean score of 4.6±1.26 (mean ± standard deviation; data are presented as such, throughout, unless otherwise stated). If treatment could improve VSS score by 1, which was considered clinically significant, approximately 26 patients per group would have been necessary to provide a result with a real significance (using the same SD and considering the standard α error of 0.05 and a power of 0.8). Assuming a 10% non-compliance rate for follow-up evaluation, the sample size was increased to 30 per group.

### Patients and Randomization

Between July 2010 and March 2012, sixty consecutive patients were enrolled for randomization having satisfied the following criteria. Inclusion criteria were: (1) adult (16 years or older) Oriental Taiwanese patients who desired elective CLSR surgery; (2) moderate-to-severe secondary scar deformity following primary cleft lip repair that warranted revision surgery; (3) valid written informed consent provided for surgery and trial inclusion. All CLSRs were performed at the Craniofacial Center of Chang Gung Memorial Hospital, Taiwan.

A specialized nurse, who was independent of the study, prepared the encoded vials. Experimental group vials contained 0.6 ml of normal saline with 15 units of botulinum toxin A (ie. 100 units of botulinum toxin A per 4 ml of normal saline; Botox, Allergen Inc, Irvine, California). Vehicle-control group vials contained 0.6 ml of normal saline. All vials appeared identical except for their randomization code and patient identification.

**Figure 2:**
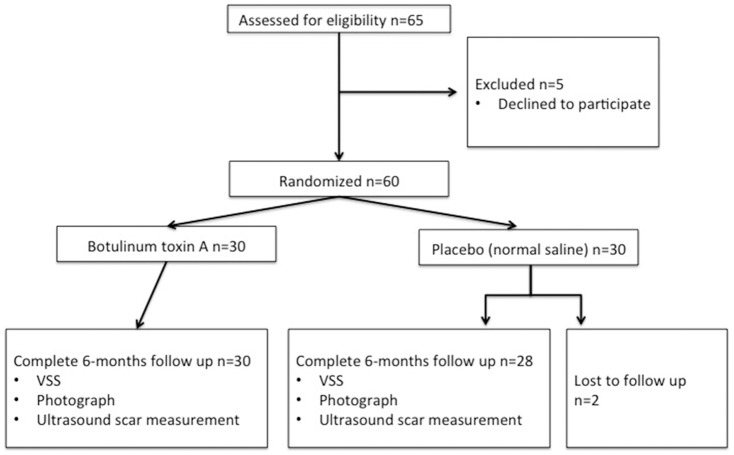
Consort statement flow chart.

Patients were block randomized to a 1∶1 ratio by an independent third-party specialized trials nurse into the experimental or vehicle-control group. Randomization codes were not revealed to anyone until after completion of the entire study. Patients, investigators, study personnel and outcomes assessors therefore remained blinded throughout the investigation (Fig. 2)(S1 Checklist).

### Secondary cheiloplasty and injections of vial contents

**Figure 3:**
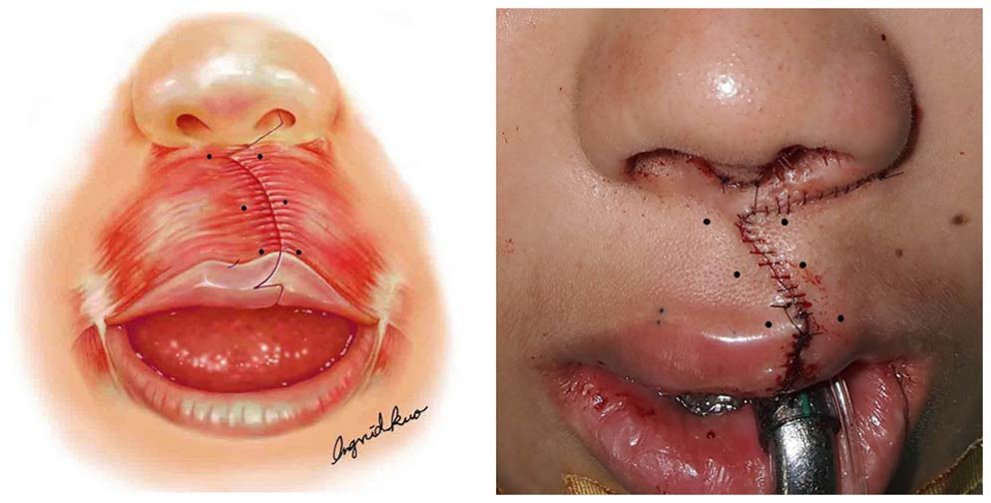
Six injections of encoded vial content were administered to the orbicularis oris muscle 5 mm either side of the wound below the nasal base and above the vermillion border.

The lip scar was revised using a modified rotation advancement cheiloplasty as previously described [8]. Briefly, the scar of the lip and nasal floor was marked and the scar tissue narrowly excised. The orbicularis oris muscle was excised wider than the width of scar; this maneuver reduces tension across the overlying skin repair, since the muscle repair causes slight redundancy of the skin. The first stitch taking lateral orbicularis muscle was sutured to the nasal septum; thereafter, the lateral orbicularis muscle was interposed and sutured overlapping the medial orbicularis muscle for philtral column reconstruction. Interrupted sutures used were as follows: 4–0 and 5–0 polydioxanone (PDS II; Ethicon/Johnson-Johnson, New Brunswick, New Jersey) for muscle and subcuticular layers, respectively; 6-0 nylon (Unik, Taipei, Taiwan) for skin [8]. Immediately after skin closure, six injections of encoded vial content (0.1 ml for each injection site) were administered to the orbicularis oris muscle 5 mm either side of the wound below the nasal base and above the vermillion border (Fig. 3).

Nylon sutures were removed six days postoperatively. To reduce wound tension caused by the adjacent risorius, zygomaticus major and zygomaticus minor muscles, both cheeks and the upper lip suture line were taped as per our routine for 6 months. Additionally, silicone sheeting was applied overnight as per our routine for 6 months. Compliance with both practices was consistent for all patients. All complications, such as hematoma, infection, wound dehiscence, oral incompetence, eating/drinking dysfunction and drug allergy, were logged if encountered.

### Records and Measurements

Primary and secondary end points were Vancouver scar scale (VSS) score (comprising the following components: pigmentation, vascularity, pliability and scar height), Visual analogue scale (VAS) and scar width, respectively. At six months follow-up, two plastic surgeons (PKTC and CSC) examined the patients in the outpatient clinic of Chang Gung Memorial Hospital. They were blinded to which group patients belonged. Scars were assessed using the VSS and assigned the mean score of the two observers.

For photographs, one standard surgical ruler was placed over the lower lip and a frontally orientated photograph of the patient was taken at 6-months follow up. The same professional craniofacial medical photographer took all photographs. Patient's photographs were subjectively assessed using VAS by five independent examiners (two Attending Plastic Surgeons and three laypersons). All examiners were both independent of the patients' care and blinded to their treatments. They were asked to score the scars on the photographs using a standard Visual Analogue Scale (VAS) graded from 0 (worst possible scar) to 10 (best possible scar).

**Figure 4:**
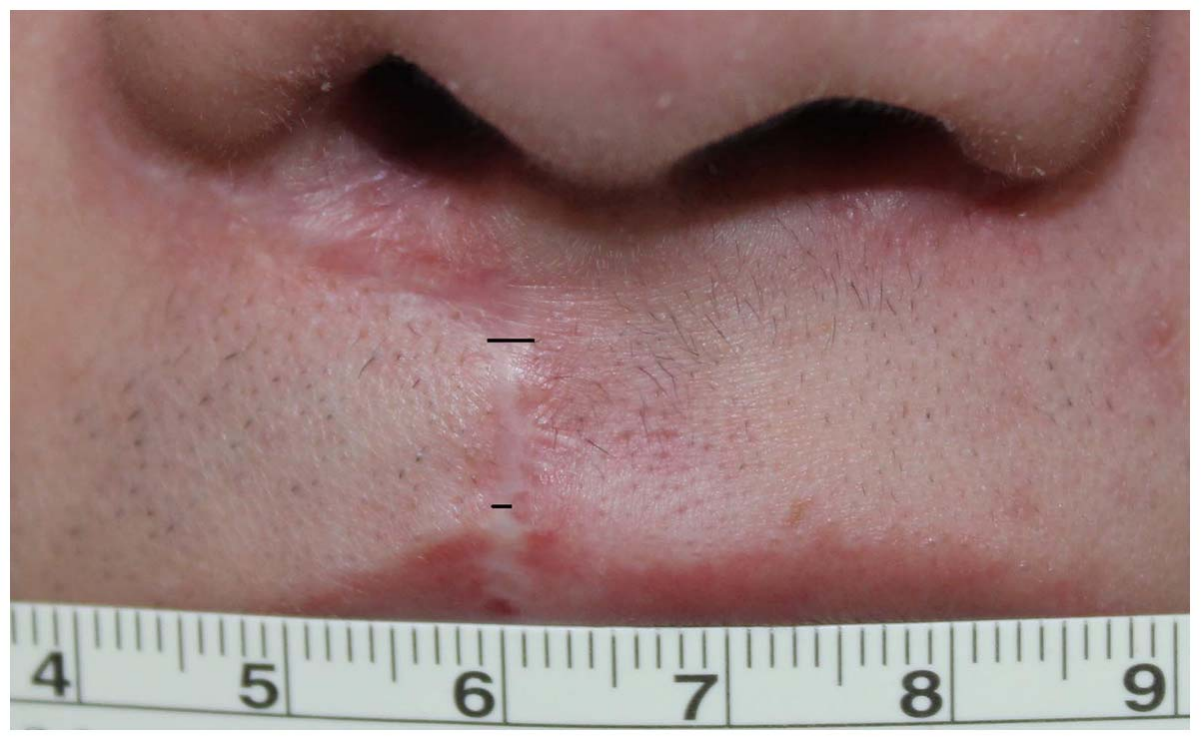
Photographic measurement of the scar: the First Point was 1 mm above the white roll and the Second Point was 1 mm below the turning incision line, close to the nasal sill.

Objective scar width measurements were performed using photography and ultrasonography. The scar was measured at two points with Photoshop (CS5 extended version 12.0; Adobe Systems Inc, San Jose, California) using the ruler as a control reference. The First Point was 1 mm above the white roll and the Second Point was 1 mm below the turning incision line, which is located close to the nasal sill (Fig. 4). The scar measurement was measured by two plastic surgeons and averaged.

**Figure 5:**
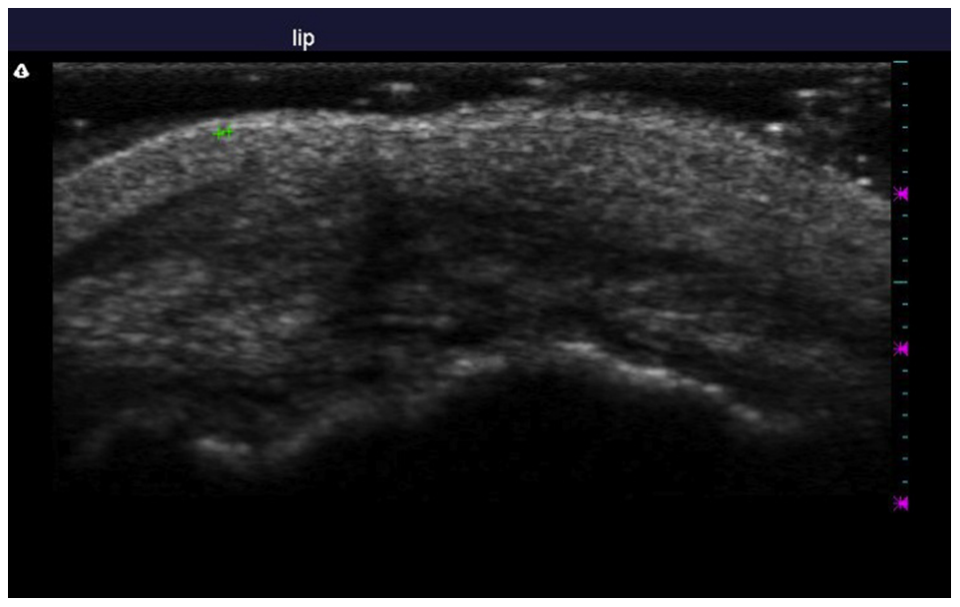
The width of upper lip scar was measured by ultrasound, as indicated by the two green crosses.

A commercial 12-MHz ultrasound transducer and imager (Model T3000; Terason, Northborough, MA, USA; settings: depth of penetration, 20 mm; capacity of ultrasound imager, 0.1 mm) was also used to quantify scar width. The transducer was placed with its upper border touching the columella-philtral junction, upper lip imaging was obtained and the scar width measured at the skin surface (Fig. 5). The ultrasound measurement was performed by the lead author (CSC) in duplicate and averaged.

### Inter- and Intra-observer consistency of assessments

Inter-observer reliability of the VSS and VAS were tested using Cronbach α. Inter-observer reliability of photographic measurements was assessed with Pearson correlation by comparing two sets of measurements performed by two independent raters. Intra-observer reliability of ultrasound measurements was assessed with Pearson correlation by comparing two sets of measurements performed by the same rater.

### Statistical analyses

All statistical analyses were conducted using SPSS software (version 17.0; IBM Corporation, NY, USA). Differences between VSS scores, VAS and between scar widths obtained photographically and ultrasonographically were compared. The independent t-test was used to compare groups. Statistical significance was defined if p was less than 0.05. Data are presented as mean ± standard deviation unless otherwise stated.

## Results

Thirty patients in the experimental group received botulinum toxin injections and thirty patients in the vehicle-control group received normal saline injections, as described, immediately after completion of CLSR. Fifty-eight patients completed six months of follow-up; two patients in the vehicle-control group failed to return for postoperative assessments. No complications were encountered (S1 Data).

The mean ages for the experimental group and vehicle-control group were 24.70±7.16 versus 21.87±8.00. There were 12 males and 18 females with 20 left and 10 right cleft lips in the experimental group. There were 14 males and 14 females with 20 left and 8 right cleft lips in the vehicle control group.

The VSS score for the experimental group was significantly lower than that of the vehicle-control group (2.45±1.52 versus 3.50±1.88; p = 0.023). Inter-observer consistency in using the VSS score was high (Cronbach α = 0.936).

The VAS score in the experimental group was significantly better than in the vehicle-control group (7.47±0.64 versus 6.10±1.06; p<0.001). Inter-observer consistency was high (Cronbach α = 0.923)

According to photographic measurements, the scar was significantly narrower at both the First Point (0.62±0.18 mm versus 0.95±0.31 mm; p<0.001) and the Second Point (0.63±0.18 mm versus 0.92±0.36 mm; p<0.001) in the experimental than in the vehicle-control group. Inter-observer consistency was high for the photographic data (First Point: r = 0.87, p<0.001; Second Point: r = 0.88, p<0.001). This was also the case according to ultrasonographic measurements (0.72±0.25 mm versus 1.03±0.42 mm for experimental and vehicle-control groups respectively; p = 0.001). Intra-observer consistency was high for the ultrasonographic data (r = 0.90, p<0.001).

## Discussion

Facial scars can be cosmetically disfiguring and may, in some patients, cause functional impairment and psychosocial withdrawal [9], [10]. Cutaneous scars are generally distinguished from surrounding normal skin by differences in color, thickness, contour, compliance, overall cosmesis and functional detriments such as contracture formation. Young et al found that patients were usually dissatisfied with their surgical scars, irrespective of patient gender, age, ethnicity or geographical location, and that 91% of them would value even a small improvement in their scar [3]. Hence, surgeons frequently recommend and prescribe scar modulation practices and treatments, particularly in susceptible ethnicities such as Orientals for whom the incidence of unfavorable scarring is higher than in Caucasians [11].

Synergistic contractions of the facial muscles are responsible for facial expressions. Since these muscles do not have bony insertions and lie very superficially, their actions exert tension across adjacent skin and subcutaneous tissue. If a wound is orientated perpendicular to the direction of the underlying facial muscle fibers, the muscular tension exerts a distracting effect on the healing wound edges and increases the risk of an undesirable hypertrophic or widened scar (Fig. 1). Consequently, reducing tension around a wound is important for improving scar quality and reducing the incidence of hypertrophic scars [12], [13].

A secondary cleft lip deformity is one that arises despite primary surgical treatment of the cleft lip. The principles of CLSR are cleft scar excision, anatomical repair of the orbicularis oris muscle, and correction of asymmetries noted in the Cupid bow and philtral columns. Unfortunately, CLSR wounds are unavoidably orientated perpendicular to the line of pull of the subjacent orbicularis oris muscle, which is in constant use during daily life for speech, eating, drinking, blowing, sucking, and a variety of facial expressions. These repetitive bouts of distracting tensional forces inflict micro-trauma to the healing wound, leading to prolongation of the inflammatory response and ultimately increased fibrosis [14]. In contrast to other facial wounds, the incidence of hypertrophic scars affecting the upper lip is high, ranging between 12% and 27% in the mixed population, and increasing when controlled for ethnicities to 32.2% in Hispanics and 36.3% in Asians. Intraoperatively, we therefore create a degree of skin redundancy with the interposing and overlapping orbicularis oris muscle repair both for philtral column restoration as well as reducing tension across the skin repair. Additionally, postoperatively we employ a routine scar management protocol in further attempts to reduce skin tension as much as possible.

Firstly, taping of the skin is commonly used for its splinting effect, which is known to improve scarring by protecting healing cutaneous wounds from tensional forces [7]. We therefore firmly tape the upper lip as well as the cheeks following CLSR routinely to counteract not only the distracting forces of the orbicularis oris but also other adjacent muscles of facial expression (such as risorius and zygomaticus major/minor). Secondly, we also apply silicone sheeting routinely overnight, given its proven utility in reducing scar hypertrophy [15]. Despite meticulous surgery and these rigid postoperative wound care practices, we are still frequently disappointed by hypertrophic scarring following CLSR. We therefore sought a new scar modulation strategy.

Botulinum toxin causes chemical muscular paralysis [16]. Function returns over several months with nerve sprouting and growth of new neuromuscular junctions. Scott found that injections of botulinum toxin could temporarily weaken extraocular muscles and used small doses to treat patients with strabismus [17]. The observation by Carruthers and Carruthers that patients treated with botulinum toxin for blepharospasm had improvements in their dynamic glabella rhytides sparked its widespread cosmetic use [18]. They showed that injections of small quantities of botulinum toxin into specific facial expression muscles caused their temporary paralysis and a concurrent reduction in overlying skin tension, thus improving dynamic wrinkles for a period of 2–6 months.

The same effect may also be exploited to reduce tension around healing facial wounds. Gassner et al demonstrated that botulinum toxin injections improved scarring from facial wounds in non-human primates [4]. Other authors have since reported apparent improvements in scars treated with botulinum toxin in humans, but their conclusions were significantly limited by the lack of controls in their studies [19]–[22]. Although Gassner et al's study was prospective, blinded, randomized and placebo-controlled, they included widely differing wounds (varying in length from 2.0 to 12.0 cm, caused by a variety of mechanisms, including traumatic lacerations and excisional cancer ablations) which were managed in non-standardized fashion (using different suture materials and suturing techniques performed by emergency physicians, otolaryngologists or plastic surgeons each with different postoperative management routines) and therefore subject to equally variable possible scar outcomes that may or may not have been directly related to the botulinum toxin or placebo [5]. Additionally, although they limited the location of wounds to the forehead, the orientation of the wounds was not considered despite it being critically important to final cosmetic outcome. Scars were only subjectively assessed using a visual analogue scale, with the added complexity that the two assessors were asked to consider the cosmetic outcome of the wound at six months with respect to their subjective opinion of the condition of the wound at closure. Furthermore, the forehead is less dynamic than the upper lip and hence at less risk of unfavorable scarring. Indeed, there is only one paper reporting two case reports of botulinum toxin injection for lower facial wounds [22]. Both wounds, however, had intrinsically favorable scarring characteristics from their outset, with the first being almost completely limited to the vermilion of the lower lip and the second being almost exactly aligned with the relaxed skin tension lines of the cheek. The benefit of botulinum toxin on scarring from both of these favorable wounds was therefore unclear and assumed.

**Figure 6:**
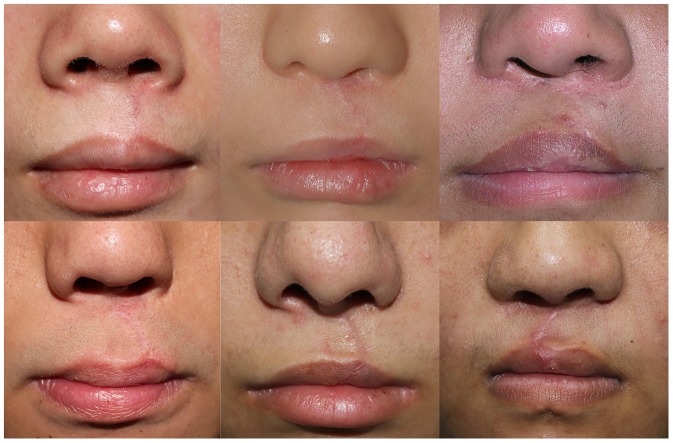
The upper row illustrates scarring of three patients in the experimental group (botulinum toxin) and lower row illustrates the scars of three vehicle-control group patients at six months' follow-up.

The present trial was therefore carefully limited to CLSR as a consistent model of lower facial wounding with a known risk of hypertrophic scarring and consistent perpendicular orientation to underlying expressive musculature. These patients and wounds were standardized in terms of ethnicity, mechanism of sharp incisional injury, CLSR design and resultant wound orientation, repair method, sutures and suturing techniques used, suture removal time, postoperative taping and silicone application, and lack of postoperative complications that affect scarring, such as dehiscence, infection and hematoma. The critical difference was the content of the randomized post-repair injections. Postoperative subjective assessments were semi-quantitatively performed using the validated VSS and VAS, and objective quantitative measurements were performed using two modalities that each presented high inter- and intra-observer consistency. Accordingly, we were able to draw a robust conclusion that the botulinum toxin injections resulted in significantly improved CLSR scars (Fig. 6), due to temporary chemical orbicularis oris paralysis and a consequent reduction in overlying skin tension forces.

Limitations of this study include, firstly, that interpretation of the absolute boundaries of a cutaneous scar is not binary and therefore may be subject to variation when performing scar width measurements. Intra-observer reliability analyses, however, were high. Secondly, since the width of a scar may not be uniform throughout its length, the points at which scars widths were measured, photographically or ultrasonographically, may not have been representative of the entire scar.

## Conclusion

This randomized, double-blinded, vehicle-controlled, prospective clinical trial demonstrated that botulinum toxin significantly improved quality of scarring following CLSR.

## Supporting Information

S1 Checklist **CONSORT checklist.**
(DOC)

S1 Protocol **Original protocol of study.**
(DOCX)

S1 Data **Raw data of study.**
(XLSX)
