# Supplementary material for: Botulinum Toxin to Improve Results in Cleft Lip Repair: A Double-Blinded, Randomized, Vehicle-Controlled Clinical Trial
Source: PLoS One. 2014 Dec 26;9(12):e115690. doi: 10.1371/journal.pone.0115690 (PMC4277415; doi:10.1371/journal.pone.0115690)
Supplement: S1 Protocol — Original protocol of study. (DOCX) [file pone.0115690.s002.docx]

**Botulinum Toxin to Improve Results in Cleft Lip Repair**

| 1. BACKGROUND   The primary correction of the unilateral and bilateral cleft lip and nasal deformities has seen great improvements over the last twenty years. With the help of presurgical orthopedics or nasoalveolar molding, surgeons have a much better condition to start with. Better understanding of the cleft pathology, evaluation of the long term results and technical refinements have all contributed to the improvement of the treatment result and therefore decrease the severity of the secondary deformities. However, the widened scar after primary surgery was still a problem (Fig 1). If the patient is unsatisfied the lip after primary surgery, secondary revision is required after the child reach adulthood. The major objectives of secondary revision or lip revision surgery are to correct the notorious widened, distorted, and prominent lip scar. Even after revision surgery, it is still difficult to predict a good outcome for the surgical scar in most patients (Fig 2).  Scars widen when opposing forces that tend to pull apart the suture line are applied to newly formed collagen before it reaches final maturity, a process that can take several months before being completed. This tensile distracting force on the upper lip is caused by the orbicularis oris muscle pull and elastic forces of the adjacent skin. The abundant motility of the lip musculature constantly pulls in different directions, not giving the collagen any rest while it matures. This may explain the occurrence of many ugly scars after lip repair (Fig 3). Botulinum toxin type A (Botox; Allergan, Inc., Irvine, Calif) is widely used for facial rejuvenation and many other medical indications. It induces chemodenervation through its action on the presynaptic neuron, preventing release of acetylcholine, which leads to functional denervation of striated muscle for 2 to 6 months after injection. The use of botulinum toxin injection to reduce the facial scar is logical because this could reduce the tensile distracting force of the upper lip caused by the orbicularis oris muscle pull. Tollefson has demonstrate that botulinum toxin injection has decrease lip tension after primary lip repair in 3 children at 3-6 months of age (Tollefson TT 2006) .  2. OBJECTIVES  The main aim of this randomized trial is to compare whether post-operative peri-surgical-injection of botulinum toxin into bilateral orbicularis oris muscles can improve scar formation for both primary and secondary cleft lip surgery. |
| --- |

表C007 共 第 第 頁

| Methods:  This is a randomized controlled trial primarily designed to compare the scarring after primary and secondary cleft lip repair using post-operative botulinum toxin injection. There will be 4 main groups in this study:-   1. Study group I: For primary lip surgery, immediately after primary lip repair will received botulinum toxin injection (1-2U/kg, at 25U/mL) into the bilateral aberrant oriented orbicularis ocuris muscle via 4 superficial injection site (Tollefson TT 2006). 2. Study group II: For revision lip surgery, immediately after revision lip surgery 3 injection of 2.5U of botulinum toxin with a distance of 0.5 cm from each injection and operative wound are injected over both sides of upper lip in a adult on the operation room. 3. Control group I : There will be the same amout of normal saline injection after primary lip surgery at 3 months of age. 4. Control group II: There will be the same amount of normal saline injection after revision lip surgery (secondary cleft lip repair).   Patients with either primary or secondary cleft lip repair  Randomize  Study group I & II:  Botulinum toxin injection  Control group I and II: Normal saline injection  Follow Up  Vancouver scar scale & ultrasound scar measurement  4. ELIGIBILITY  4.1 Inclusion criteria for the primary lip repair group  (i) Baby born with cleft lip who will receive primary lip repair at 3 months of age  (ii) Written informed consent given by parent/guardian.  (iii)For the cleft gap ≤ 5mm the patient is classified as mild cleft lip, for cleft gap > 5 mm the patient is classified as moderate-severe cleft lip.  4.2 Inclusion criteria for the secondary lip repair group  (i) Adult > 16 years old.  (ii) Moderate to severe secondary cleft lip and/or nose deformity that warrants corrective surgery.  (iii) Written informed consent given by parent/guardian/patient.  4.3 Patient numbers: 60 for primary lip repair and 60 for secondary lip repair  4.31 Sample size calculation:  10 consecutive patient selected from our OPD on march 2009.  Vancouver Scar Scale means 4.6 with SD 1.264911 (rage 3-7). If the study group with improvement of 1 is clinical significant, giving power 0.8 with the same SD, the sample size calculated will be 26.  Using Terason Ultrasound (capacity of measured 1/100 cm or 1/10 mm) the scar width mean is 1.13 mm with SD .6201254. If the study group with improvement of 0.5 mm as clinical significant, giving the power of 0.8 with the same SD, the sample size calculated will be 25.  We will use 30 patients for each group.  4.41 Exclusion criteria for primary lip repair  a. Combined other craniofacial anomalies  b. Without permission of parent/guardian, without signed informed consent by parent/guardian.  4.42 Exclusion criteria for the secondary lip repair  a. Less than 15 years old  b. Mild secondary cleft lip and / or nose deformity that does not warrant corrective surgery.  c. Without written informed consent.  d. Without permission of parent/guardian, the patient signed the consent himself.  5. Botulinum Toxin injection  5.1 For Study group I:  Injections are placed into the orbicularis oris muscle 5 mm adjacent to the scar and the vermilion border on each side of the operative wound (A total of 3 injections per side making a total of 6 injections per patient) (Fig 4). 1-2U/kg (baby) and 2.5 U (adult ) of botulinum toxin (Botox; allergen, Inc., Irvine, Calif) will be used for each injection. A total dosage of 15 U will be injected into each patient.  5.2 For Control group:  Injection of the same amount of normal saline at the same place will be performed.  6. STUDY VISITS AND ASSESSMENTS  6.1 Registration and Randomization  (i) Confirmation of eligibility  (ii) Information Sheet  (iii) Written informed consent  (iv) Registration of baby and adult into study and randomization  (v) Scheduling of patient for botox injection immediately after surgery.  6.2 Follow-up Assessments  6.2.1 **Vancouver scar scale (Sullivan T 1990)** measures pigmentation, vascularity, pliability and scar height on the postoperative 6 month F/U. (Table 1)  Ultrasonography measurement of the scar (Fong SS 1997)  For both primary and secondary lip surgery, the patient will receive postoperative follow up during 1wk, 1mo, 3mo, 6mo. On the 1st week postoperative follow up, the stitches will be removed, 3M taping will be placed over the wound to reduce surface tension and minimize the scar formation. On the 6th mo follow up, a lay person will assess the scar using the Vancouver scar by the same surgeon (Chun-Shin, Chang) and a plastic surgeon will use ultrasonography to measure the scar. Each patient will be measured 3 times by the same doctor (Chun-Shin, Chang)  The Independent T-Student test will be used to analyze the statistical significance between the two groups.  7. Expected result  In this study we expect that 70% of study group will have better lip scar and narrower lip scar compared to the control group in both primary and secondary lip repair group.  8. Possible insult  The dosage of Botox that we use is very tiny. Systemic insult is unlikely to happen. If an allergic reaction occurred it will be treated according to the severity of allergy and Diphenhydramin PO or IV form or systemic corticosteroid will be given.  Reference:  Fong SS, H. K., Chen JC (1997). "The cutometer and ultrasonography in the assesment of post burn hypertrophic scar: A preliminary study." Burns **23 (Suppl.1)**: S12.  Sullivan T, S. J., Kermode J (1990). "Rating the burn scar." J Burn Care Rehabil **11**: 256-260.  Tollefson TT, S. C., Sykes JM (2006). "Botulinum Toxin to Improve Results in Cleft Lip Repair." Arch Facial Plast Surg **8**: 221-222.  Figures:  Figure 1  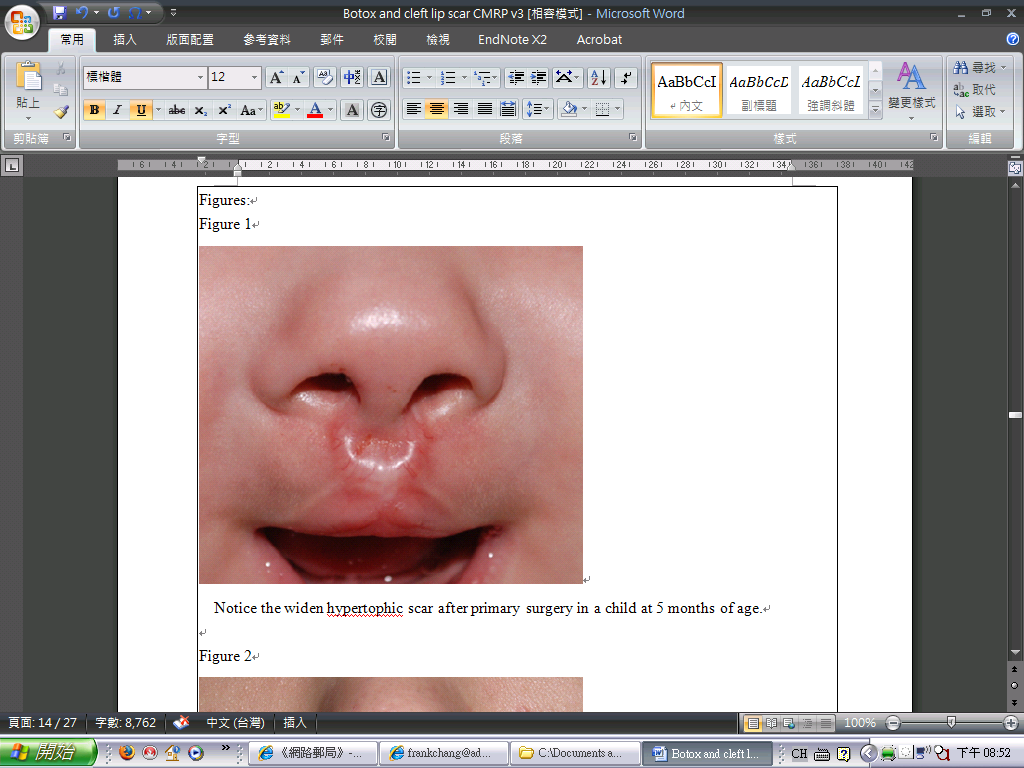  Notice the widen hypertophic scar after primary surgery in a child at 5 months of age.  Figure 2  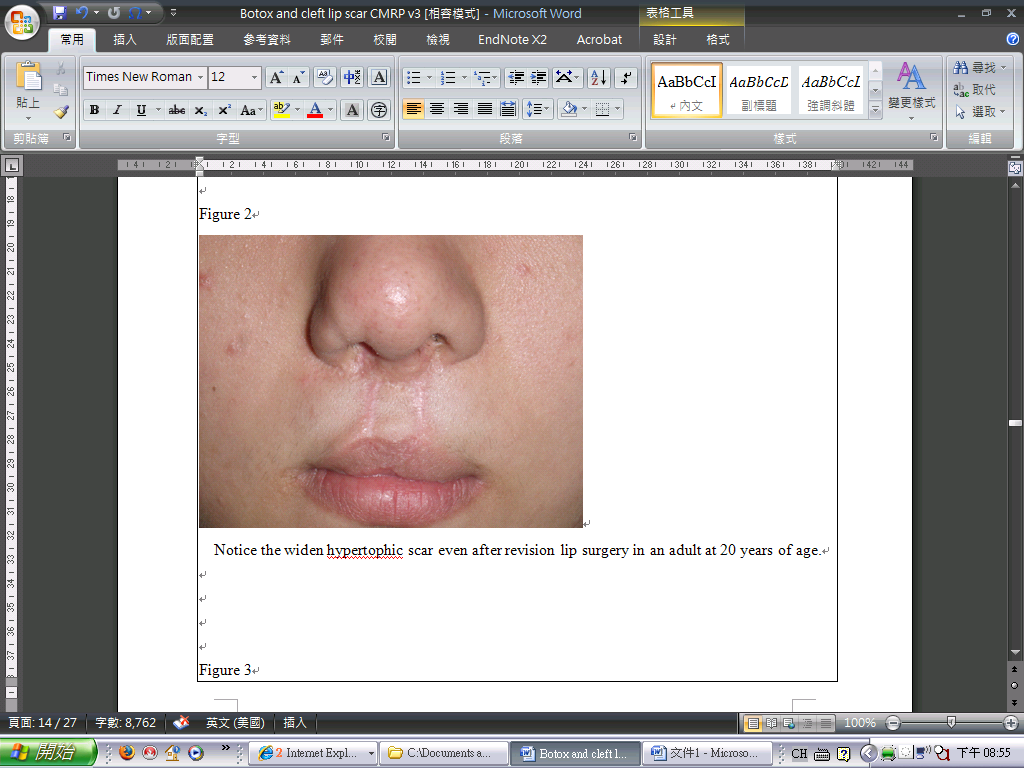  Notice the widen hypertophic scar even after revision lip surgery in an adult at 20 years of age.  Figure 3  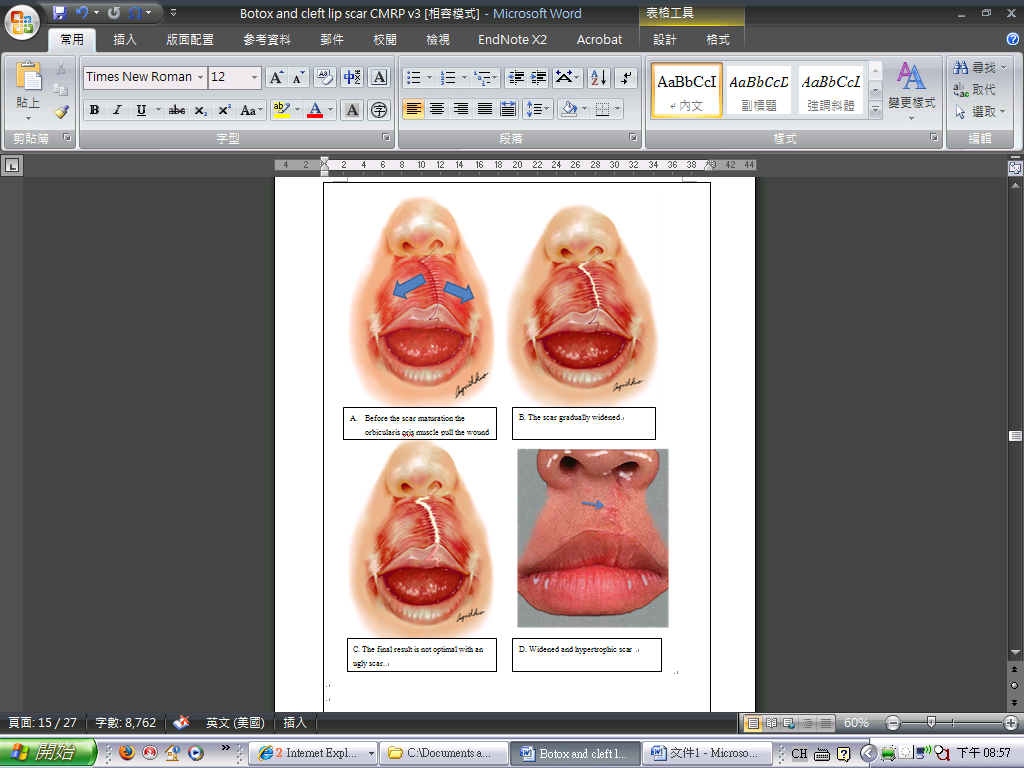  D. Widened and hypertrophic scar  C. The final result is not optimal with an ugly scar.  B. The scar gradually widened   1. Before the scar maturation the orbicularis oris muscle pull the wound   Figure 4  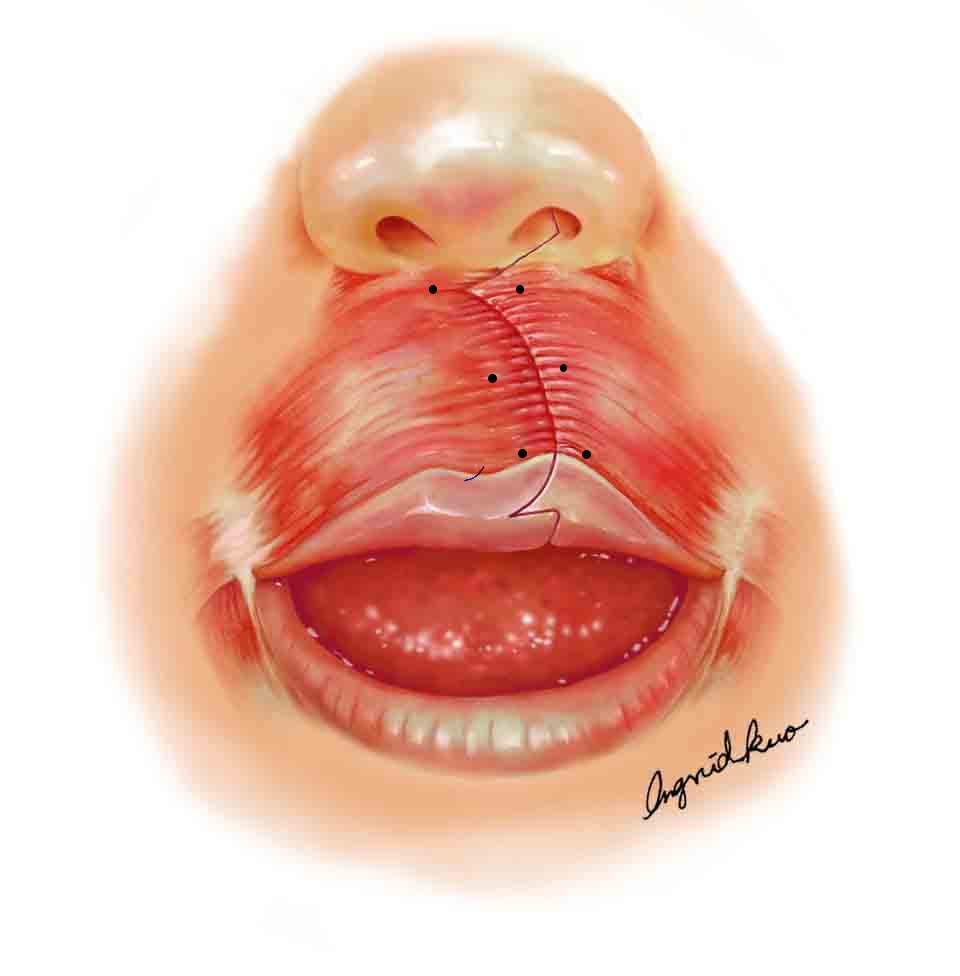  Table 1  Scar Assessment Tools:   1. **Vancouver scar scale(Sullivan T 1990)**   measures pigmentation, vascularity, pliability and scar height.  Pigmentation  0 normal: color that closely resembles the color of the rest of the body  1 Hypopigmentation  2 Hyperpigmentation  Vascularity  0 normal: color that closely resembles the color of the rest of the body  1 pink  2 red  3 purple  Pliability  0 normal  1 supple: flexible with minimal resistance  2 yielding: giving way to pressure  3 firm: inflexible, not easily moved, resistant to manual pressure  4 banding: rope-like tissue that blanches with extension of the scar  5 contracture: permanent shortening of the scar producing deformity or distortion  0 Normal: flat  1 < 2 mm  2 < 5 mm  3 > 5 mm |
| --- |
